# Microbial food webs share similar biogeographic patterns and driving mechanisms with depths in oligotrophic tropical western Pacific Ocean

**DOI:** 10.3389/fmicb.2023.1098264

**Published:** 2023-01-26

**Authors:** Qianwen Shao, Dong Sun, Chen Fang, Yunzhi Feng, Chunsheng Wang

**Affiliations:** ^1^Key Laboratory of Marine Ecosystem Dynamics, Second Institute of Oceanography, Ministry of Natural Resources, Hangzhou, China; ^2^Ningbo Institute of Oceanography, Ningbo, China; ^3^Southern Marine Science and Engineering Guangdong Laboratory (Zhuhai), Zhuhai, China; ^4^College of Oceanography, Hohai University, Nanjing, China; ^5^School of Oceanography, Shanghai Jiao Tong University, Shanghai, China

**Keywords:** tropical western Pacific Ocean, microbial food web, interspecies interaction, biogeographic pattern, driving mechanism, North Equatorial Current

## Abstract

Microbial food web (MFW) dominates the energy flow in oligotrophic tropical open ocean pelagic ecosystems. Understanding biogeographic patterns and driving mechanisms of key components of the MFW is one of the central topics in current marine ecology. Investigations were conducted along an 1,100-km horizontal gradient and in the full-water column vertical gradient of the oligotrophic tropical western Pacific Ocean. High-throughput sequencing and association networking methods were used to analyze the community structure and interspecies interactions of MFW. The structure of MFW significantly differed with depths, but not across horizontal gradients. Bacteria and microeukaryotes were interconnected and had more predominantly positive and negative linkages in the aphotic layers. Key components of MFW exhibited similar biogeographic patterns and driving mechanisms. Geographic distance exerted minimal effects on the distribution patterns of the microbial food web, while environmental factors played more important roles, especially for temperature and inorganic nutrients. Stochastic processes were more important in the microbial food webs of the 5–200  m layer than the >500  m layer, and drift explained the majority of stochastic processes. Moreover, only a weak but not significant driving force for North Equatorial Current on the east–west connectivity of the microbial food web was found in the upper layers. This knowledge is a critical fundamental data for future planning of marine protected areas targeting the protection of tuna fishing in the western Pacific Ocean.

## 1. Introduction

The significance of microbial food web (MFW) in oligotrophic ocean was originally proposed by Pomeroy (1974). Azam et al. (1983) formalized the concept of the microbial loop; then, Sherr and Sherr (1988) broadened the definition of the microbial loop to include direct trophic links between phytoplankton and phagotrophic protozoa, in addition to the multi-step detritus-bacteria flagellate-ciliate pathway. Currently, MFW mainly consists of bacteria, archaea, *Synechococcus*, *Prochlorococcus*, picoeukaryotes, microzooplankton (heterotrophic and mixotrophic nanoflagellates and ciliates) and planktonic viruses, which are key groups that control nutrient cycling and biomass production in aquatic ecosystems, especially oligotrophic oceans (Seymour et al., 2017). In the MFW, different species are directly and indirectly linked through biogeochemical cycles and food web interactions. Heterotrophic bacteria and phytoplankton are interconnected by a two-way flux: phytoplankton excretion or cell lysis is a source of organic matter for heterotrophic bacteria and the mineralization of this organic matter by heterotrophic bacteria in turn provides nutrients to primary producers (Sarmento et al., 2010). Protists (e.g., nanoflagellates, ciliates) are grazers in the MFW, which are strictly heterotrophic or mixotrophic, and incorporate carbon by ingesting small primary producers and/or heterotrophic bacteria (Zubkov and Tarran, 2008). A correlation network analysis revealed that sequencing data can be used to hypothesize positive (mutualism and symbiosis) and negative (competition and parasitism) ecological interactions between various species (Faust and Raes, 2012). To date, only a few studies have investigated the MFW in oligotrophic oceans (Calbet and Landry, 1999).

The tropical western Pacific Ocean is one of the world’s largest warm oligotrophic regions, which has been largely unexplored with regard to the diversity and biogeography of planktonic microbiome, based on Tara Oceans data (Pesant et al., 2015). Only a few studies have used micro-optical based techniques, such as microscopy and flow cytometry, to study the community structure and biomass of phytoplankton (Dai et al., 2020; Wei et al., 2020), picoplankton, and virioplankton in the surface layer and vertical changes in the MFW around seamounts in the oligotrophic tropical western Pacific Ocean (Liang et al., 2017; Zhao et al., 2020; Wei et al., 2021). The development of high-throughput sequencing technology over the last decade has greatly improved our understanding of planktonic prokaryotes and eukaryotes in the oligotrophic tropical western Pacific Ocean (Wang et al., 2020; Wu et al., 2020; Zhao et al., 2020; Shao et al., 2022). Compared to optical based methods, it can be used to identify variations in less abundant species and picoplankton populations of the MFW. The tropical western Pacific Ocean is important tuna fishing ground (Worm et al., 2005), but primary production cannot be passed up to these large predators without the transfer of MFW (Calbet, 2008; Zeldis and Décima, 2020). Knowledge about the geographical patterns and driving mechanisms of MFW is therefore a critical fundamental data for future planning of marine protected areas targeting the protection of these large predators. In addition, we rely heavily on this knowledge to assess the impacts of climate change on pelagic ecosystems in open ocean (Pinsky et al., 2020).

One of central topics in current marine ecology is to elucidate the biogeographic patterns and driving mechanisms of marine microorganisms. Knowledge of the underlying mechanisms that shape microbial biogeographic patterns is necessary for understanding the community structure and to exercise best practices in the stewardship of marine resources (Zhou and Ning, 2017). However, most studies have focused on eutrophic and mesotrophic offshore and coastal environments (Djurhuus et al., 2020; Richter et al., 2020); only a few have investigated oligotrophic oceans. Previous studies demonstrated that environmental factors (temperature, light, and nutrients) and spatial factors significantly correlated with the distribution of bacteria and microeukaryotes in the oligotrophic tropical western Pacific Ocean (Wang et al., 2020; Wu et al., 2020; Zhao et al., 2020; Shao et al., 2022), indicating that environmental selection-related processes are more important than dispersal-related processes in shaping the distribution of bacteria and microeukaryotes. However, the proportion of bacterial and microeukaryotic community variations explained by both processes is small, which implies that the complex distribution patterns within communities and other drivers (currents, water mass, and biotic interactions) may significantly contribute to shaping the distribution of MFW in the oligotrophic tropical western Pacific Ocean. Null model analyses can be used to assess the importance of different drivers of bacterial and microeukaryotic community assembly in the oligotrophic tropical western Pacific Ocean (Kong et al., 2022; Xu et al., 2022), including dispersal limitation, homogenizing dispersal, heterogeneous selection, homogeneous selection, and drift (Stegen et al., 2013). The strong Pacific North Equatorial Current (NEC) is the main influencing current of tropical western Pacific Ocean, providing favorable conditions for east–west connectivity in the upper (0–200 m) western Pacific between 8 and 18°N (Qiu and Chen, 2012; Hsu et al., 2017). However, the importance of the NEC in shaping the MFW in the oligotrophic tropical western Pacific Ocean remains poorly understood.

In this study, the spatial variation and driving factors of bacterial and microeukaryotic communities in the oligotrophic tropical western Pacific Ocean were analyzed to enhance our understanding of the biogeographic patterns and assembly mechanisms of the MFW. In this extremely oligotrophic environment, we hypothesized that: (i) bacteria and microeukaryotes have different predominant linkages with depths; (ii) bacterial and microeukaryotic communities share similar biogeographic patterns and assembly mechanisms; and (iii) the NEC enhances the east–west connectivity of the MFW in the upper tropical western Pacific Ocean.

## 2. Materials and methods

### 2.1. Sampling site and collection

**Figure 1:**
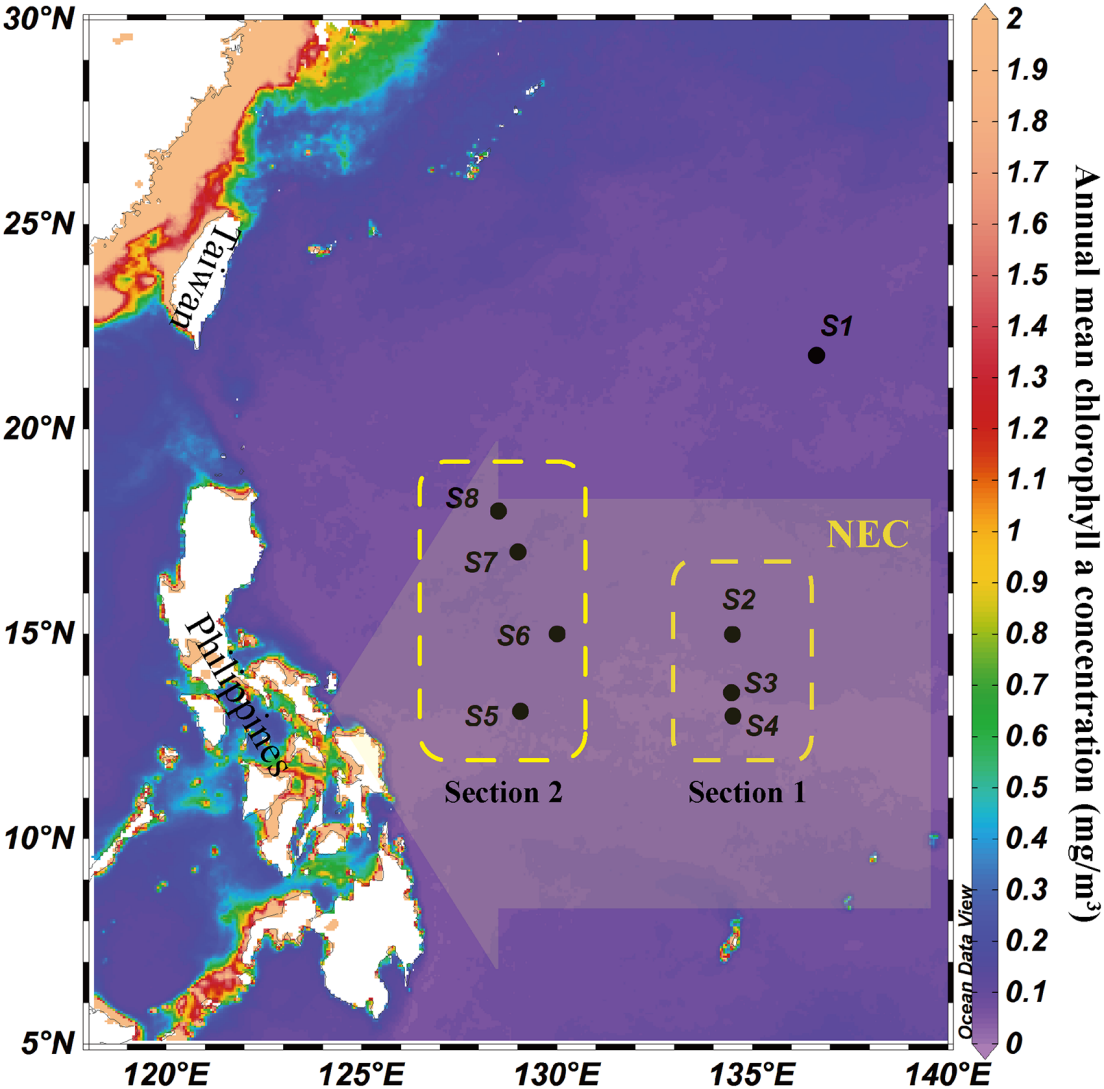
Location of eight water column sampling sites (black circles) and schematic illustration of the influence range of North Equatorial Current (NEC, yellow arrow) in the western tropical Pacific Ocean. Yellow boxes indicated two sections in the NEC influence area. The background was the annual mean chlorophyll a concentration (mg/m^3^).

Sampling sites and sample collection were processed and analyzed following previously described methods (Shao et al., 2022), which were consistent between microeukaryotes and bacteria (Figure 1). Depths of eight stations ranged from 1,629 to 5,648 m, and we collected samples from six different depths (5 m, 75 m, deep chlorophyll maximum (DCM), 200 m, 500 m, and 3,000 m) at each station. The annual mean chlorophyll a concentration (mg/m^3^) was downloaded from the E.U. Copernicus Marine Service, which indicated that the sampling area was a typical oligotrophic sea. Eight sampling sites, except S1, were located in the influence area of the Pacific NEC (130–160°E, 8–18°N).

### 2.2. DNA extraction, PCR amplification, and Illumina sequencing

DNA extraction was conducted using a DNeasy Power Soil Kit (QIAGEN, Valencia, CA, United States) according to the manufactures instructions. The concentration and purity of the total DNA were measured by a NanoDrop ND-100 spectrophotometer (Thermo Fisher Scientific, Wilmington, DE, United States). Primers 547F (5’-CCAGCASCYGCGGTAATTCC-3′) and V4R (5’-ACTTTCGTTCTTGATYRA-3′) were used to amplify the V4 region of 18S rRNA (Stoeck et al., 2010). Adapter sequences were linked to the barcodes at the 5′ end of each primer. Microeukaryote PCR amplification, purification, and quantification were conducted following the same methods as for bacteria (Shao et al., 2022). High-throughput gene sequencing was performed on an Illumina Novaseq-PE250 platform (paired-end reads, 2 × 300 bp) by Personal Biotechnology, Co., Ltd., (Shanghai, China).

### 2.3. Sequence assembly, clustering, and annotation

The quality filter and assembly of raw microeukaryote sequence were the same as for bacteria, which followed previously described methods (Shao et al., 2022). High-quality representative sequences for amplicon sequence variants (ASVs) were assigned with 100% sequence identity using UCLUST (Edgar, 2010). Annotation was carried out using UCLUST and the National Center for Biotechnology Information (NCBI) gene database. The ASVs affiliated with chloroplasts, mitochondria, unclassified groups, and singletons were removed from the dataset. To correct possible errors induced by unequal sequencing efforts in all samples, the ASV table was randomly subsampled to ensure an equal number of sequences per sample (16,704) using MOTHUR v1.33.3 (Schloss et al., 2009). The α-diversity indices, including Pielou’s evenness, Faith’s phylogenetic diversity, and Shannon-Wiener and Simpson indices, were calculated using QIIME v2.0.0 (Caporaso et al., 2010). Good’s coverage was calculated using MOTHUR v1.33.3 (Schloss et al., 2009).

### 2.4. Statistical analyses

All statistical analyses were performed using R v4.1.1 software,^1^ unless noted otherwise. Prior to statistical analysis, the ASV table was Hellinger-transformed and the environmental variables were normalized using the “vegan” R package to improve normality and homoscedasticity (Oksanen et al., 2007).

The group compositions of the microeukaryotic communities with relative abundances >1% were visualized at the phylum and order levels. Bacterial taxonomic groups with relative abundances >1% were visualized at the order level. The α-diversity indices were visualized using the “ggplot2” R package (Gómez-Rubio, 2017). Wilcox tests were used to evaluate differences in the α-diversity indices between the six depths using the “ggpubr” R package (Kassambara, 2020). Venn diagrams were used to show the microeukaryotic relationships among the six depths using the “VennDiagram” R package (Chen and Boutros, 2011). The β-diversity was calculated using Bray-Curtis distances and visualized with a principal coordinated analysis (PCoA) ordination. Linear discriminant analysis effect size (LEfSe) was used to screen potential statistically significant microeukaryotic taxa with total abundances >10 among the six depths. The non-parametric factorial Kruskal-Wallis sum-rank test (alpha value = 0.05) was used to detect taxa with significantly different abundances between groups. A linear discriminant analysis (threshold score = 4.0) was used to estimate the effect sizes of each abundant taxa (Segata et al., 2011).

A network analysis was calculated and visualized using Cytoscape v3.9.1. First, the orders of bacteria and microeukaryotes with average relative abundance of less than 1% were removed, and a Spearman rank correlation matrix was created using the remaining data. Then, co-occurrence patterns were determined if the Spearman’s correlation coefficient was >0.4 (positive) or < −0.4 (negative) and *p* < 0.05 (Zhou et al., 2018). To test the effects of the NEC on the MFW, subsequent analyses were divided into 5–200 m and > 500 m layers of the bacterial and microeukaryotic communities. To study distance-decay relationships in the MFW, the relationship between the Bray-Curtis dissimilarities of bacterial and microeukaryotic communities and geographic distances was analyzed based on Spearman’s rank correlations. A permutational multivariate analysis of variation (PERMANOVA) was applied to partition the variations of all, 5–200 m, and > 500 m layers of bacterial and microeukaryotic community compositions among different depths, stations, and sections. Sections were classified by different latitudes in the NEC influence area (Figure 1; Supplementary Figures S2–S4 were one section, and Supplementary Figures S5–S8 were another section). A set of spatial factors was generated using the procedure coordinates of neighbor matrices (PCNMs) method based on the longitudes and latitudes of the sampling stations (Borcard and Legendre, 2002). Mantel tests were conducted to investigate the sources of bacterial and microeukaryotic variation while considering both spatial and environmental factors (Diniz-Filho et al., 2013). Redundancy analysis and Spearman’s rank correlation were used to explore the effects of environmental variables on the MFW (Oksanen et al., 2007). Then, we used mantel tests and variation partitioning analysis to quantify the relative effects of environmental and spatial factors in shaping the MFW following previously described methods (Shao et al., 2022).

To evaluate the relative effects of stochastic and deterministic processes, phylogenetic structures of the bacterial and microeukaryotic communities were used to obtain insights into the drivers of community assembly. We assessed the relative importance of heterogeneous selection, dispersal limitation, drift, homogenizing dispersal, and homogeneous selection using a three-step framework (Stegen et al., 2013). First, the intercommunity phylogenetic turnover was calculated using the mean nearest taxon distance (βMNTD). Then, the β-nearest taxon index (βNTI) was estimated, which represents the difference between the observed βMNTD and mean null expectation in units of standard deviation (999 randomizations). βNTI > +2 indicated heterogeneous selection, and βNTI < −2 indicated homogeneous selection. Finally, the Bray-Curtis-based Raup-Crick metric (RC_bray_) was calculated, which provided insight into the contribution of stochastic processes to community assembly, when −2 < βNTI < +2. RC_bray_ < −0.95 indicated homogenizing dispersal, −0.95 < RC_bray_ < +0.95 indicated drift, and RC_bray_ > +0.95 indicated dispersal limitation. Randomization was performed 999 times. The averages (%) were subsequently calculated, as they indicated the relative importance of different ecological processes.

## 3. Results

### 3.1. Environmental factors of the water columns

Supplementary Figure S1 showed the highest temperatures were in surface water and decreased gradually to the lowest depth at 3,000 m. Salinity was highest in the DCM and lowest at 5 m. Dissolved oxygen increased from 5 m to 75 m, and then volatility decreased at 500 m and 3,000 m. The concentrations of chlorophyll an increased from 5 m to the DCM, then sharply decreased at 200 m and were reduced to zero in the deeper layers. The concentrations of nutrients (total inorganic nitrogen, dissolved inorganic phosphate and silicate) continuously increased from 5 m to 3,000 m depth. More details were described in Shao et al. (2022).

### 3.2. Community structure of MFW with depths

High-quality sequence numbers of each microeukaryotic sample ranged from 21,172 (S2-200) to 89,829 (S7-3000), with an average of 60,637. Good’s coverage ranged from 97.17% (S8-500) to 99.77% (S7-3000 and S8-3000), with an average of 98.98%. The 16,007 ASVs from 43 samples were obtained, and the ASV number in each sample ranged from 176 (S4-3000) to 1,396 (S8-500), with an average of 699 (Supplementary Table S1). Regarding the distribution patterns of all ASVs in different layers (Supplementary Figure S2), 63 ASVs (0.39%) occurred in all depths, while the total unique ASVs were 2,552, 2,045, 1,561, 2,569, 3,524, and 1,052 in the 5 m, 75 m, DCM, 200 m, 500 m, and 3,000 m layers, respectively.

**Figure 2:**
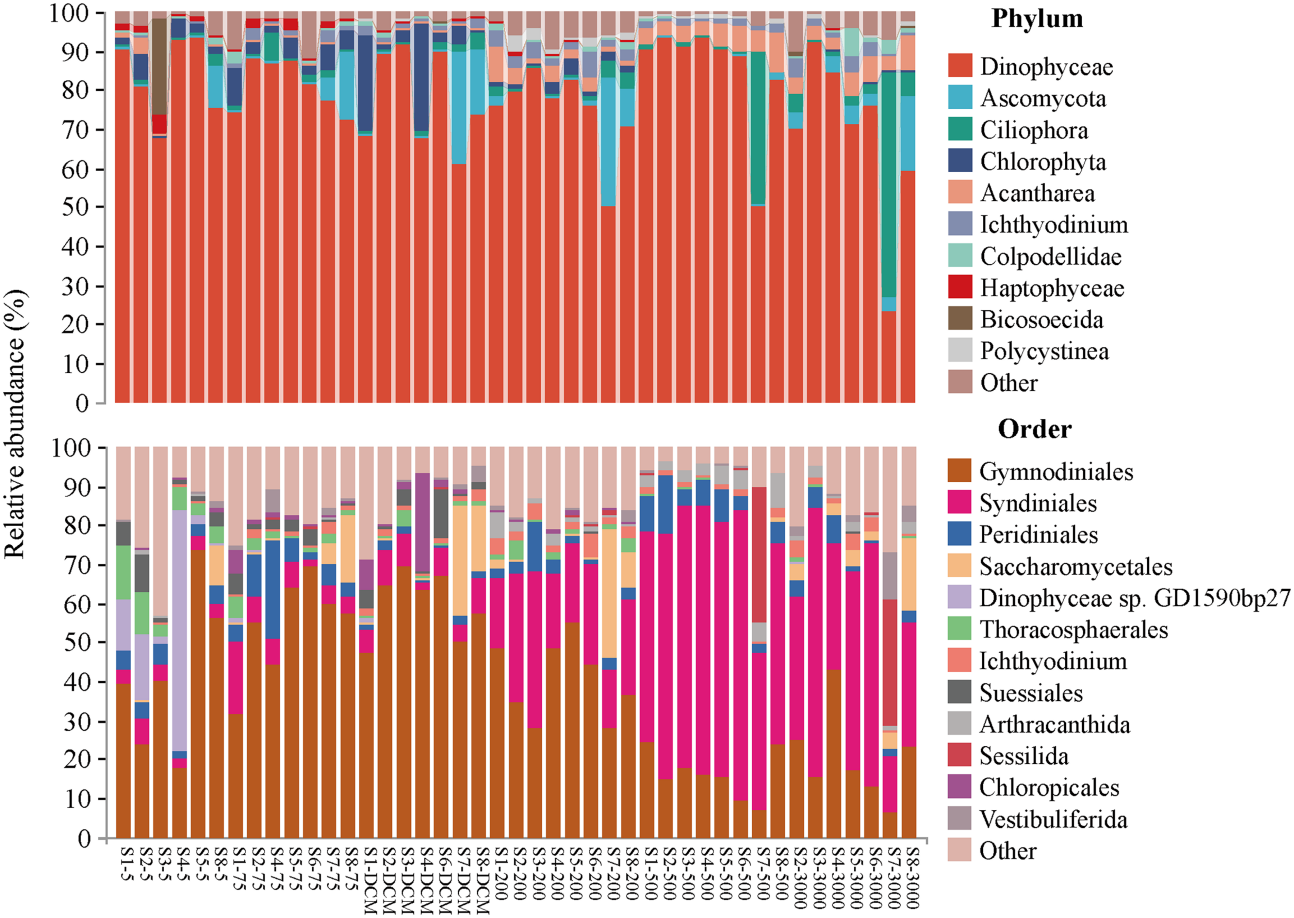
Relative abundance of microeukaryotic taxonomic groups at the phylum (top 10 phyla and others) and the order (relative abundance of  > 1%) level across 43 samples.

The relative abundances of the microeukaryotic communities in different depths exhibited different patterns (Figure 2). Dinophyceae was the most abundant microeukaryotic phylum in the investigation area, accounting for more than half of the proportion of the community in almost all samples, except S7-3000. Dinophyceae mainly consisted of the orders Gymnodiniales and Syndiniales. Gymnodiniales contributed more to relative abundance in the 5–200 m layer, while Syndiniales showed the opposite trend. The other top microeukaryotic phyla included Ascomycota (3.99%), Ciliophora (3.91%), Chlorophyta (3.08%), Acantharea (2.80%), and Ichthyodinium (1.78%), which had relative abundances >1%. The other top microeukaryotic orders included Peridiniales (4.69%), Saccharomycetales (3.87%), and Dinophyceae sp. GD1590bp27 (2.29%), which had relative abundances >2%.

The α-diversity indices of the microeukaryotes in the photic layers showed little fluctuation, which were higher in the 200-and 500-m layers than in the 300-m layer among the aphotic layers. Based on the Wilcox tests, the α-diversity indices of samples in the DCM significantly differed from that of samples in the other layers, while no significant differences were detected between the other layers (Supplementary Figure S3). As per the β-diversity analysis, the PCoA ordinations based on the ASV distributions across the 43 samples indicated that the microeukaryotic community shifted across the six depths (Supplementary Figure S4). LefSe analysis was used to identify differentially abundant taxa among the six depths categorized by microeukaryote successional dynamics (Supplementary Figure S5). The abundances of order Gonyaulacales, and genus *Xiphacantha* (including species *Xiphacanthaalata*), were higher in the 5 m layer. The classes, Polycystinea and Spirotrichea, significantly differed in the 200 m layer, while classes, Acantharea and Conidasida (including order Eucoccidiorida), had high abundances in the 500 m layer. The abundance of Oligohymenophorea in the 3,000 m layer was significantly higher than that in the other layers.

The taxonomic compositions of bacteria and their relative abundances are shown in Supplementary Figure S6. At the order level (relative abundances >2%), bacterial sequences were mainly assigned to Betaproteobacteriales (17.48%), Nitrosopumilales (12.81%), Clostridiales (10.73%), Synechococcales (9.30%), SAR11 (7.34%), Bacteroidales (4.11%), Lactobacillales (3.72%), Rhodospirillales (2.85%), Caulobacterales (2.44%), Microtrichales (2.35%), Flavobacteriales (2.31%), and SAR202 (2.18%). Synechococcales were mainly found in the photic zone. In contrast, Nitrosopumilales were mainly found in the aphotic zone. More details of bacterial community structure and α-diversity with depths were described in Shao et al. (2022), which found the 200 m layer had the highest Pieloou’s evenness, Faith’s phylogenetic diversity, Shannon–Wiener and Simpson indices, followed by the DCM layer and 500 m layer, and lowest in the 5-and 75-m layers.

### 3.3. Co-occurrence networks of bacteria and microeukaryotes in the MFW

**Figure 3:**
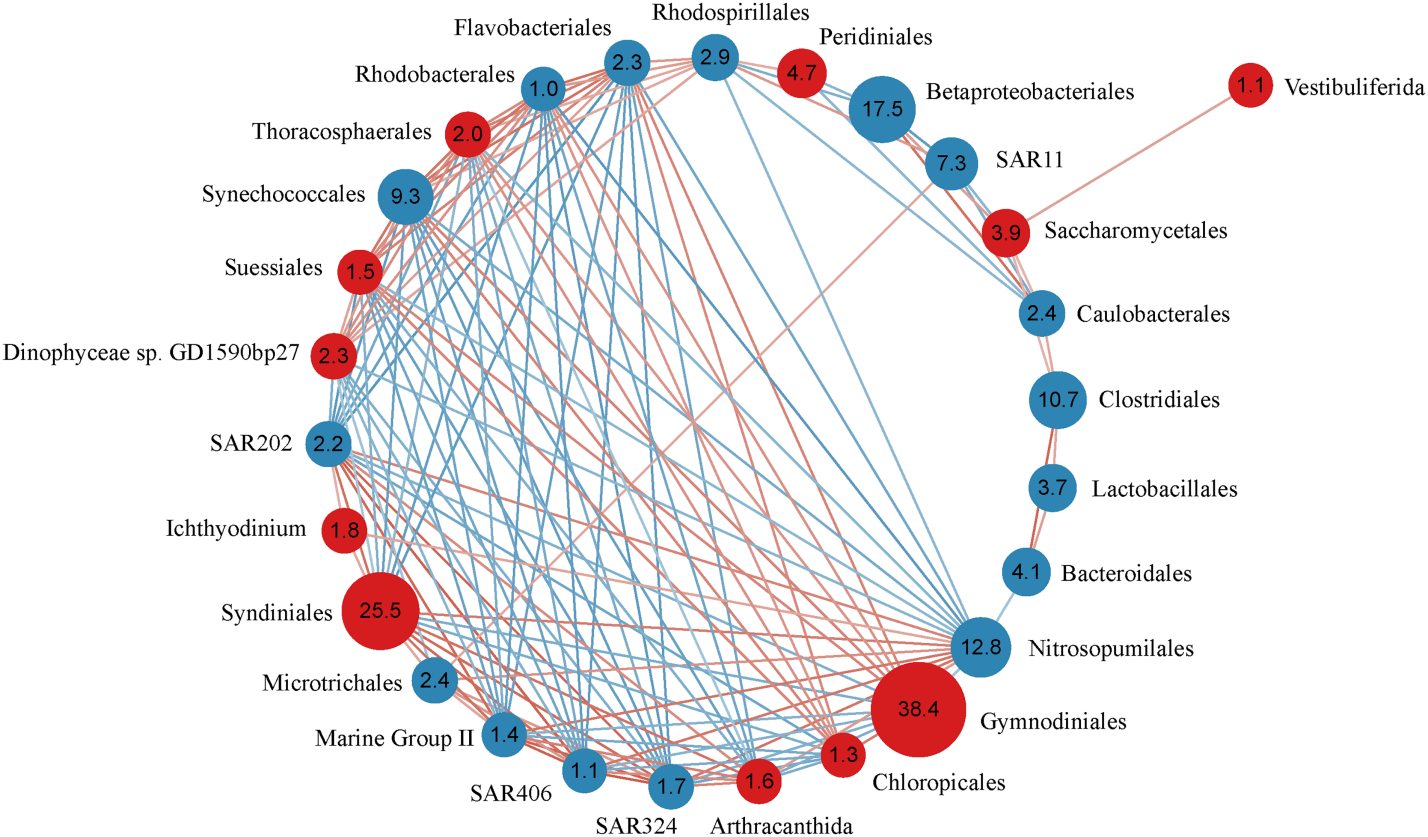
Correlation relationship with the order (relative abundance of >1%) comprising bacteria and microeukaryotes across 43 samples. The network shows strong (*R* = 0.4) and significant (*p* < 0.05) Spearman rank correlation coefficients. Red circles represent microeukaryotic orders, blue circles represent bacterial orders. Numbers in the circles are the relative abundance of bacterial/microeukaryotic orders. Red lines indicate positive correlations, blue lines indicate negative correlations.

The Spearman correlation analysis revealed strong correlations between 16 bacteria and 11 microeukaryote orders, except Sessilida (Figure 3). All microeukaryotic groups correlated with bacteria, except Vestibuliferida. Some bacteria specifically associated with other bacteria. For example, the bacteria SAR 324, SAR 406, SAR202, and Marine Group II highly positively correlated with each other (*R* > 0.87), as well as positively correlated with the microeukaryotes Syndiniales (*R* > 0.81) and Arthracanthida (*R* > 0.64), but negatively correlated with the bacteria Rhodobacterales (*R* < −0.75), Flavobacteriales (*R* < −0.74), and Synechococcales (*R* < −0.71) and the microeukaryotes Suessiales (*R* < −0.69), Chloropicales (*R* < −0.64), Thoracosphaerales (*R* < −0.63), and Dinophyceae sp. GD1590bp27 (*R* < −0.60). Moreover, Rhodobacterales, Flavobacteriales, and Synechococcales highly positively correlated with each other (*R* > 0.81) and positively correlated with the microeukaryotes Suessiales (*R* > 0.68), Thoracosphaerales (*R* > 0.67), and Gymnodiniales (*R* > 0.60).

Significant correlations were detected between different microeukaryotic groups. For example, Syndiniales highly positively correlated with Arthracanthida (*R* = 0.81). Both microeukaryotes negatively correlated with Gymnodiniales, Suessiales, and Chloropicales (*R* < −0.64). Suessiales and Chloropicales positively correlated with Gymnodiniales and Thoracosphaerales (*R* > 0.64). Vestibuliferida only positively correlated with Saccharomycetales.

### 3.4. Distance-decay relationships in MFW

**Figure 4:**
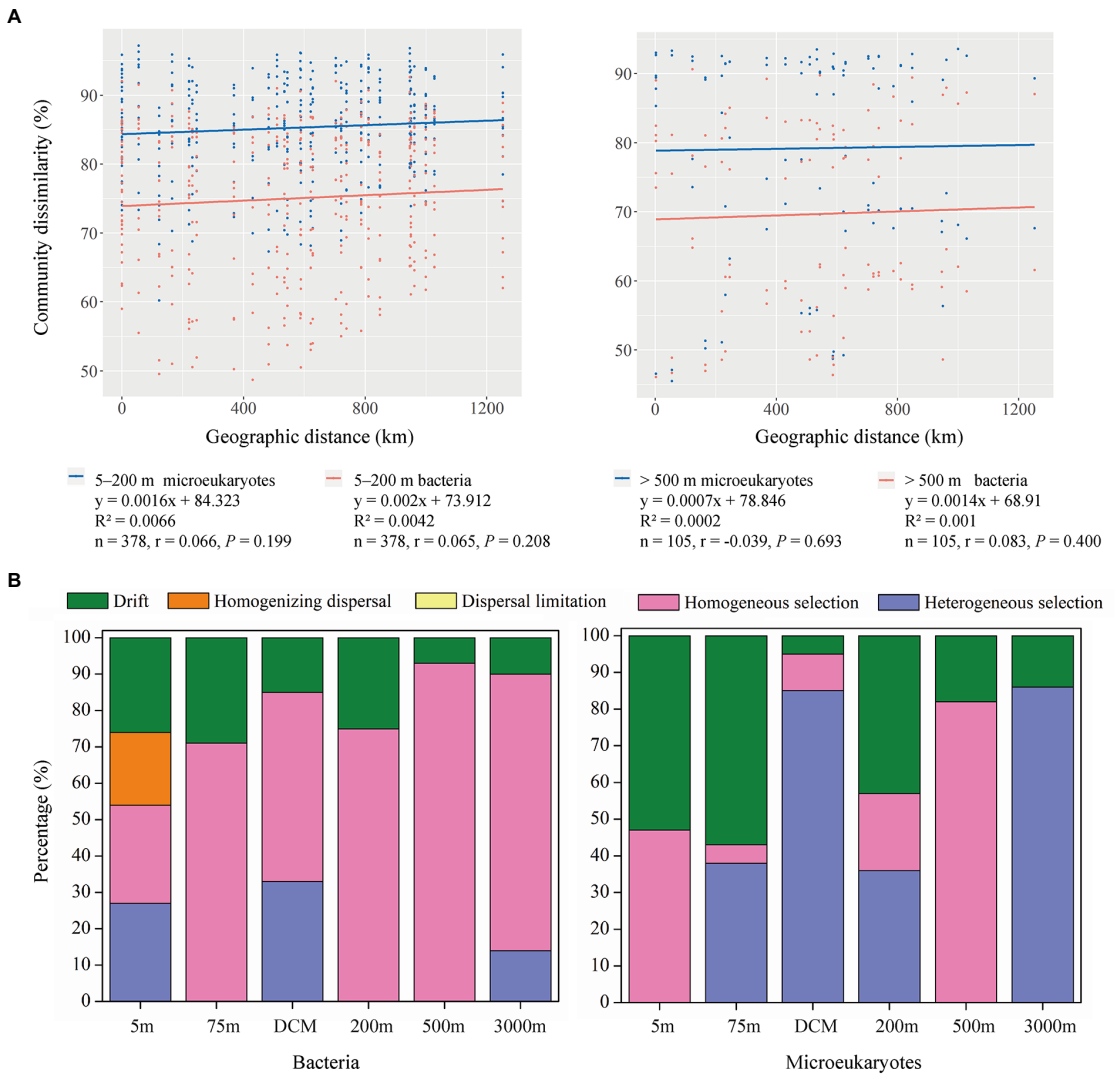
**(A)** Spearman’s rank correlations between the Bray–Curtis dissimilarity of microeukaryotic (blue dots and line) and bacterial (red dots and line) communities and geographic distance in the 5–200  m and > 500  m layers, the *n* is the number of comparisons. **(B)** Relative importance of ecological processes in shaping the bacterial and microeukaryotic communities across six depths.

Spearman correlations comparing Bray-Cutis community dissimilarities with geographic distances between samples revealed no significant positive correlations for microeukaryotic or bacterial communities in 5–200 m and > 500 m layers (Figure 4A). The PERMANOVA tests showed that all, 5–200 m, and > 500 m bacteria and microeukaryotes significantly differed among the six depths. No significant differences were detected across the eight stations and two sections analyzed herein (Supplementary Table S2).

Null model analyses detected higher levels of stochastic processes (dispersal limitation, homogenizing dispersal, and drift) in the 5–200 m layer than in the >500 m layer (Figure 4B). Drift explained the majority of stochastic processes in bacterial (18.67% on average) and microeukaryotic (31.67% on average) community turnover, while homogenizing dispersal only affected (20.00%) bacterial community turnover in the 5 m layer. Among the deterministic processes, homogeneous selection explained 65.67% of bacterial community turnover on average, and it was the most important ecological process, followed by heterogeneous selection, which explained 12.33% of bacterial community turnover. Compared to bacterial community turnover, the relative importance of heterogeneous selection increased in microeukaryotic community turnover (40.83% on average), while homogeneous selection was lower (27.50% on average).

### 3.5. Driving mechanisms of MFW

Environmental and spatial factors associated with bacterial community were processed and analyzed following previously described methods (Shao et al., 2022). For the microeukaryotic community, the redundancy analysis showed that temperature, salinity, chlorophyll a concentration, and dissolved oxygen concentration were the controlling factors associated with population variation in the 5–200 m layer, while the strongest determinants of community structure in the >500 m layer were silicate and total inorganic nitrogen (TIN), including nitrite, nitrate, and ammonium (Supplementary Figure S7). The Mantel tests indicated that the bacterial and microeukaryotic community structures significantly correlated with almost all environmental factors, especially temperature, TIN, dissolved inorganic phosphate (DIP), and silicate, which exhibited almost no significant effects with five spatial factors (PCNMs, nos. 1–5), except for the 5–200 m bacterial and microeukaryotic communities (Supplementary Table S3).

**Figure 5:**
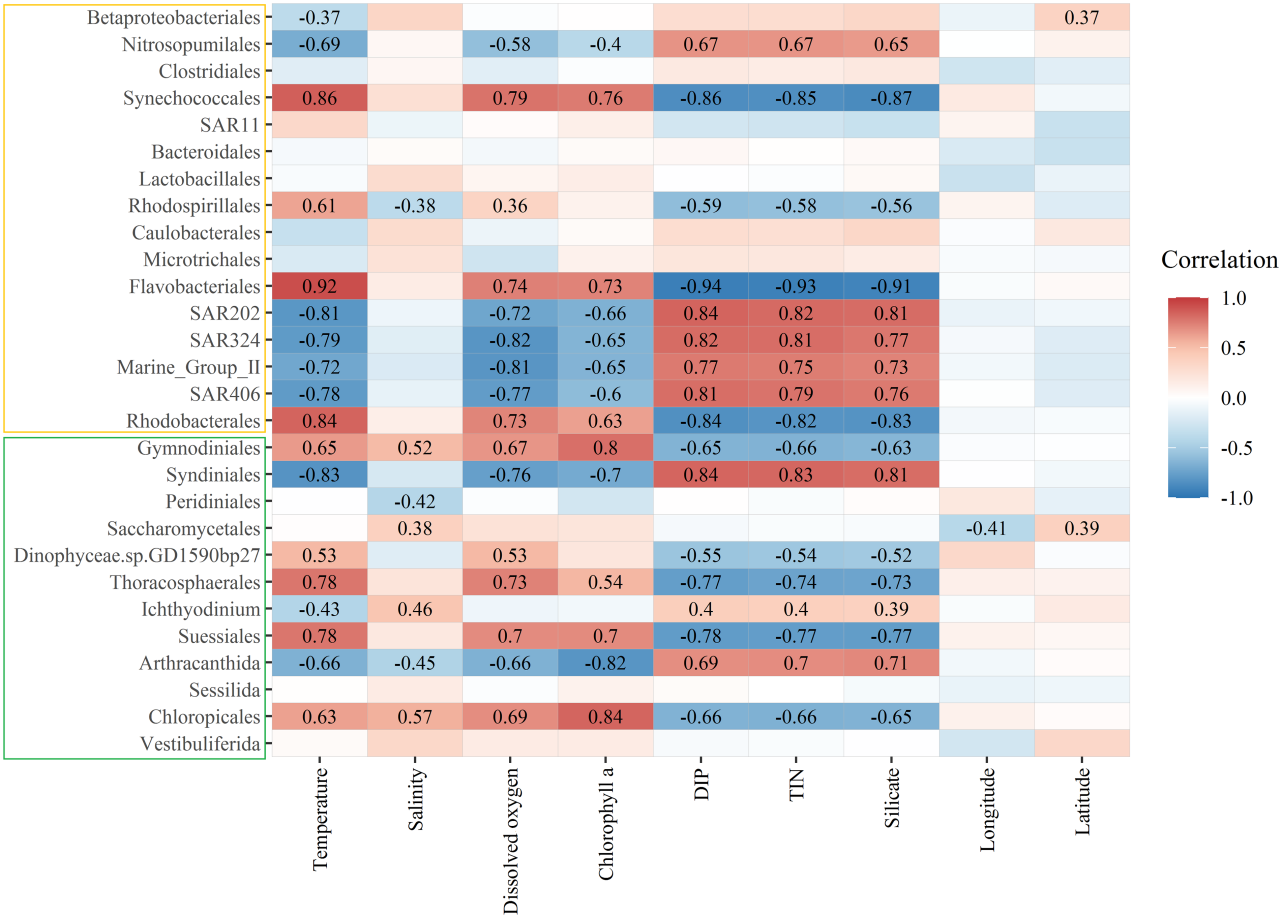
Heatmap depicting Spearman’s rank correlations between the relative abundances of main bacterial (yellow box) and microeukaryotic (green box) orders and environmental factors. Significant correlation coefficient values were marked in the figure. DIP, dissolved inorganic phosphate; TIN, total inorganic nitrogen.

The heatmap results showed that the bacteria Nitrosopumilales, Synechococcales, Flavobacteriales, SAR202, SAR324, Marine_Group_II, SAR406, and Rhodobacterales and the microeukaryotes Gymnodiniales, Syndiniales, Thoracosphacrales, Suessiales, Arthracanthida, and Chloropicales significantly correlated with temperature, dissolved oxygen, chlorophyll a concentration, DIP, TIN, and silicate (Figure 5). The bacteria Rhodospirillales and the microeukaryotes Gymnodiniales, Peridiniales, Saccharomycetales, Ichthyodinium, Arthracanthida, and Chloropicales significantly correlated with salinity, while the bacteria Betaproteobacteriales and the microeukaryote Saccharomycetales significantly correlated with longitude and latitude. Some microorganisms, like the bacteria Clostridiales, SAR11, Bacteroidales, Lactobacillales, Caulobacterales, and Microtrichales and the microeukaryotes Sessilida and Vestibuliferida, did not significantly correlate with the environmental factors examined in this study.

**Figure 6:**
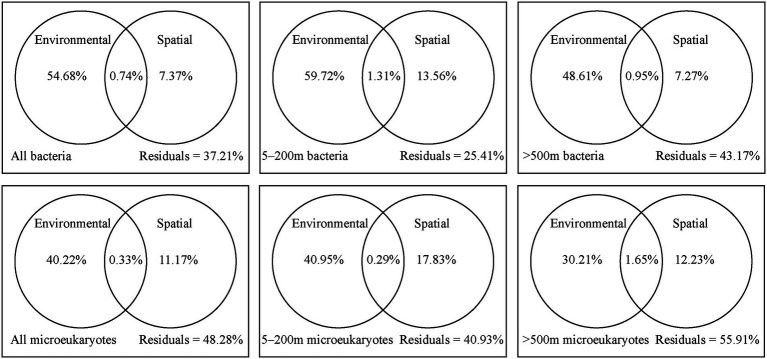
Variation partitioning analysis of all, 5–200  m, and  > 500  m bacterial and microeukaryotic community composition between environmental and spatial factors.

The variation partitioning analysis showed that environmental effects had a relatively higher contribution on the bacterial and microeukaryotic communities than spatial factors (Figure 6). Comparatively, bacteria were more affected by environmental factors, while microeukaryotes were more affected by spatial factors. For the 5–200 m layer, spatial and environmental factors collectively explained 74.59% of bacterial community variation and 59.07% of microeukaryotic community variation, and remaining variations were unexplained. Spatial factors explained 13.56% of bacterial community variation and 17.83% of microeukaryotic community variation, while environmental factors explained 59.72% of bacterial community variation and 40.95% of microeukaryotic community variation. For the >500 m layer, environmental and spatial explanatory factors for the bacterial and microeukaryotic community variations both decreased; more variations remain unexplained.

## 4. Discussion

### 4.1. Community diversity and distribution of MFW

Community diversity and distribution of bacteria had been discussed in Shao et al. (2022). In microeukaryotes, Dinophyceae were the most abundant group detected in this study (Figure 2), which was consistent with a previous study on the global ocean scale (de Vargas et al., 2015). Zhao et al. (2020) and Wu et al. (2020) also reported that dinoflagellate was the dominant group in the oligotrophic tropical western Pacific Ocean, while its operational taxonomic unit (OTU) proportion was much lower in aphotic communities than in photic communities. Dinoflagellates comprise both photosynthetic and heterotrophic flagellates, of which, 58% are heterotrophic species in the ocean (Gómez, 2012). Their adaptation to a wide range of environments is reflected by tremendous morphological and trophic diversity (Tailor, 1987). Of these, Gymnodiniales had a higher relative abundance in the 5–200 m layer, while a higher proportion of parasitic taxa, such as Syndiniales, was mainly detected in the aphotic layers (Figure 2). Gymnodiniales are mixotrophic non-thecate dinoflagellates that are generally surface-dwelling. Their appearance in deep waters is likely a result of the amplification of resting cysts, which is as an adaptive strategy for dinoflagellates to survive adverse conditions (Anderson, 1984). Syndiniales are ubiquitous marine parasite taxa that are widely distributed throughout the ocean (Anderson and Harvey, 2020). Our finding supports the hypothesis that microeukaryotic parasites may play key roles in the deep-sea food web (Zoccarato et al., 2016).

In this study, distinct vertical patterns of the microeukaryotic communities along the water column were observed in the oligotrophic tropical western Pacific Ocean (Supplementary Figure S4). Similar patterns were found for the bacterial communities (Shao et al., 2022). Both bacteria and microeukaryotes had their highest α-diversity indices detected in the 200-and 500-m layers (Supplementary Figure S3), wherein dramatic transitions occurred due to temperature, salinity, dissolved oxygen, chlorophyll a concentration, and major nutrients (Supplementary Figure S1). Different taxonomic groups exhibited various vertical patterns, which may be due to their biological characteristics and feeding habits. For instance, the diversity of Radiolarians, including Polycystinea and Acantharea, was higher in the aphotic layers than in the photic layers (Figure 2), and were biomarkers for the 200-and 500-m layers, respectively (Supplementary Figure S5). This may be partially attributed to their intracellular siliceous (Polycystinea) or celestite (Acantharea) skeletons consisting of strontium sulphate (SrSO_4_), which can thus benefit from transporting and preserving these organisms in deeper layers (Burki et al., 2010). A previous study found that the high diversity of Radiolarians in deep layers may include both local and resident populations, as well as taxa settling from the overlying water, which have been laterally advected from elsewhere (Xu et al., 2017). Ciliophora, mainly including the orders Sessilida and Vestibuliferida, appeared abundantly in the 500-and 3,000-m layers (Figure 2). The class Oligohymenophorea was the biomarker for the 3,000 m layer (Supplementary Figure S5). Sessilida are peritrichs of the class Oligohymenophorea, which are among the most speciose and commonly observed ciliates with >50 genera representing >1,000 species (Li et al., 2008). Vestibuliferida of the class Litostomatea have been reported to be found in the guts of several marine surgeonfish in the oligotrophic tropical western Pacific Ocean, as observed *via* microscopy (Grim et al., 2002). Some members of Oligohymenophorea and Litostomatea have been suggested to possess hydrogrnosome, belonging to obligatory anaerobes, and other members are facultative anaerobes that have been reportedly found in different suboxic and anoxic conditions (Lynn, 2008), which may explain their distributions in this study.

### 4.2. Interspecies interactions in the MFW with depths

Recent studies have indicated that interspecies interactions, such as mutualism, cross-feeding, competition, parasitism, predation, and allelopathy, in the MFW are responsible for community structure (Lima-Mendez et al., 2015). In our study, dinoflagellates positively correlated with specific bacterial groups and had the widest selection, where Vestibuliferida (Ciliophora) had almost no correlation with other bacteria or microeukaryotes (Figure 3). Most ciliophora are free-living, some are epi-and endo-commensals, parasites of other unicellular organisms and metazoans, and serve as hosts for bacterial epi-and endo-symbionts, fungi, algae, and other protists (Dziallas et al., 2012), like the ciliophoran Vestibuliferida and the fungus Saccharomycetales.

A previous study demonstrated that phytoplankton had a stronger relationship with bacteria than environmental factors (Pearman et al., 2016). Specific interactions between phytoplankton and bacteria have been documented, many of which are based on the exchange of energy sources and metabolites, including various types of chemical signaling (Ramanan et al., 2016). In photic layers, the bacteria Synechococcales, Flavobacteriales, and Rhodobacterale were highly positively correlated with the dinoflagellates Suessiales (*R* > 0.68), Thoracosphaerales (*R* > 0.67), and Gymnodiniales (*R* > 0.60; Figure 3). The bacterial communities that assemble around marine microphytoplankton are predictably dominated by Rhodobacterales, Flavobacteriales, and families within Gammaproteobacteria (Ferrer-González et al., 2021). A genome analysis of Flavobacteriales isolates identified several genes that encode CAZyme glycoside hydrolases (Fernández-Gomez et al., 2013). Taylor et al. (2014) proposed that Flavobacteriales were degrading and utilizing phytoplankton-derived transparent exopolymer particles. Rhodobacterales are primarily members of the Marine Roseobacter Clade (MRC), which is a metabolically diverse and opportunistic bacterioplankton group that is known to form close associations with phytoplankton blooms (Newton et al., 2010). Synechococcales (phylum Cyanobacteria) are ubiquitously distributed and are the most abundant photosynthetic organisms on Earth (Flombaum et al., 2013). Cyanobacterial symbionts (cyanobionts) have been found in numerous protist groups, including dinoflagellates, tintinnids, radiolarians, amoebae, diatoms, and haptophytes (Foster and Zehr, 2019). The relationships between cyanobionts and protistan hosts are particularly notable, as some nitrogen-fixing cyanobacteria (diazotrophs) play important roles in primary production, especially in nitrogen-limited oligotrophic oceans (Falkowski et al., 1998). However, the nature of cyanobiont-dinoflagellate host consortia remains poorly understood, our findings offer insights into these vacancies in the oligotrophic tropical western Pacific Ocean.

In the aphotic layers, the parasitic dinoflagellate Syndiniales highly positive correlated with the bacteria SAR324, SAR406, and SAR202, and Marine_Group_II (Figure 3; *R* > 0.81). Positive associations between parasites and heterotrophic bacteria may indicate that cell lysis from eukaryotic parasitism or predation leads to the release of dissolved organic matter, which in turn drives bacterial activity, similar to viral shunt (Jephcott et al., 2016). Members of SAR 324, SAR406, and SAR202 are typical free-living bacteria that serve extremely important roles in the mesopelagic and bathypelagic zones of all oceans (Salazar et al., 2016). Marine_Group_II Euryarchaeota are commonly connected with Syndiniales in oxygen-depleted water columns (Suter et al., 2021). However, each of these bacteria are described only through their ecological distributions or metagenome-assembled genomes and lack cultured representatives, making their functional characterization a challenge. Moreover, Arthracanthida (an order of Radiolarians) also highly positively correlated with the dinoflagellate Syndiniales (*R* = 0.81; Figure 3), which may result in the highly positive correlations with the bacteria SAR324, SAR406, SAR202, and Marine_Group_II (Figure 3; *R* > 0.64). Spero and Angel (1991) reported the mutualistic symbionts associated with the plankton rhizarian (Acantharia, Foraminifera, and Radiolaria) and the dinoflagellate genera *Amphidinium*, *Aureodinium*, *Gloeodinium*, *Gymnodinium*, *Gyrodinium*, *Prorocentrum*, *Pyrocystis*, and *Scrippsiella*. Our results expand this range of symbionts.

### 4.3. Factors that regulate the MFW

Generally, dispersal limitation and environmental heterogeneity are the main factors that shape the distributions of microorganisms, which generate a negative correlation between community similarity and geographic distance (Hanson et al., 2012). However, our study showed that geographic distance had little effect on the dispersal of bacterial and microeukaryotic communities over a spatial scale of 1,100 km in the oligotrophic tropical western Pacific Ocean (Figure 4A). The Tara Oceans dataset reported that community dissimilarity significantly positively correlated with geographic distance for all organismal size fractions over a transect of ~6,000 km (De Vargas et al., 2015). Several conceptual frameworks and modeling studies have demonstrated that the dispersal limitation of microbes may lead to a distance-decay relationship, while high dispersal ability weakens the relationship (Hanson et al., 2012). In our study region, Hsu et al. (2017) reported that the core of the NEC (defined as where the westward velocity is >15 cm s^−1^) occupied a region from 8 to 12.5°N and from 0 to 200 m. To quantitatively analyze the effects of the NEC on the dispersal activity of microorganisms in the six depths, we calculated the contribution of ecological processes to the assembly of MFW in the 5–200 and > 500 m layers. Higher levels of stochastic processes (dispersal limitation, homogenizing dispersal, and drift) were detected in the 5–200 m layer than in the >500 m layer (Figure 4B), which may weakly support our hypothesis that the NEC enhanced the dispersal ability and east–west connectivity of the MFW in the photic layers. Dispersal limitation had a weak effect on the MFW (Figure 4B), which was supported by the variation partitioning analysis, that is, spatial processes explained ~10% of the variance in all, 5–200 m, and > 500 m bacterial and microeukaryotic community composition (Figure 6). Drift explained the majority of stochastic processes (Figure 4B), which was the prevalent community-structuring mechanism in unicellular eukaryotes, and was lower important in structuring prokaryotic organisms (Logares et al., 2018). Some reports found that drift played a moderate role in the assembly of picoeukaryotes and prokaryotes over large spatial scales, such as those present in tropical and subtropical surface oceans (Logares et al., 2020). For deterministic processes, bacterial communities were mainly explained by homogeneous selection, while microeukaryotic communities were mainly explained by heterogeneous and homogeneous selection (Figure 4B). The shallow distance-decay relationships supported the dominance of homogeneous selection (Figure 4A), considering that homogeneous selection contributed to high community similarities (Dini-Andreote et al., 2015). Our results were in accordance with the findings of Logares et al. (2020) and Kong et al. (2022), who found that heterogeneous selection was more important in shaping microeukaryotic communities than bacterial communities, especially in the 3,000 m layer (Figure 4B). They speculated that the differences between the effects of selection may be explained by different modes of adaptation of bacteria and microeukaryotes. Moreover, the dispersal abilities of aquatic organisms and the distances they travel are poorly understood for entire biotas (Bohonak and Jenkins, 2003). In our study, only the bacteria Betaproteobacteriales and the microeukaryote Saccharomycetales significantly correlated with longitude and latitude (Figure 5), which indicated that these microorganisms are weak dispersing organisms (Heino et al., 2015). The fungi Saccharomycetales mainly appeared around stations S7 and S8 (Figure 2), which indicated the presence of organic matter (Korajkic et al., 2015).

In our study, environmental factors contributed more than spatial factors (Figure 6). Almost all environmental factors significantly correlated with the bacterial and microeukaryotic communities (Supplementary Table S3), which indicated that environmental selection played a more important role in shaping the MFW than dispersal limitation in the oligotrophic tropical western Pacific Ocean. Among these factors, temperature had the greatest effect on community, followed by inorganic nutrients (Supplementary Figure S7; Supplementary Table S3). Temperature is an important factor that alters community composition and diversity and is a stronger driver than other environmental factors in shaping microbial community composition, as per a global investigation (Sunagawa et al., 2015). Inorganic nutrients are essential for the growth and development of microorganisms, and different microorganisms adapt to their optimal growth concentrations (Follows and Dutkiewicz, 2011). Due to ocean warming, it is likely that the oligotrophic oceans will be more stratified (Sarmento et al., 2010). Vertical exchange of nutrient between nutrient-deficient upper waters and nutrient-rich deep waters will be reduced as a result of this enhanced stratification in the oligotrophic tropical western Pacific Ocean (Abirami et al., 2021). For different taxonomic groups (Figure 5), the bacteria Nitrosopumilales, SAR202, SAR324, Marine_Group_II, and SAR406 and the microeukaryotes Syndiniales and Arthracanthida negatively correlated with temperature, dissolved oxygen, and the chlorophyll a concentration and positively correlated with inorganic nutrient concentrations, while the bacteria Synechococcales, Flavobacteriales, and Rhodobacterales and the microeukaryotes Gymnodiniales, Thoracosphacrales, Suessiales, and Chloropicales exhibited opposing trends for environmental factors. These results were consistent with interspecies interactions in the MFW (Figure 3), which indicated that the adaptive capability of different taxonomic groups to environmental factors is an important factor that determines the community distribution.

Major bacterial and microeukaryotic groups were more or less regulated by environmental factors in our study (Figure 5), except for the bacteria Clostridiales, SAR11, Bacteroidales, Lactobacillales, Caulobacterales, and Microtrichales and the ciliophoran Sessilida and Vestibuliferida. Members of the SAR11 clade are the most abundant and ubiquitous bacterioplankton found in the oceans worldwide (Yooseph et al., 2010). The relative abundances of Bacteroidales, Clostridiales, and Lactobacillales were determined by the total ammonia and free ammonia concentrations (De Vrieze et al., 2015), which concentrations were not measured in our study. Ciliophora are a free-living group with cilium that are distributed in various habitats and are capable of adapting to different environments (Foissner et al., 2007). Broad niche widths and high ecophysiological tolerances of ciliates are reflected by a low limitation of dispersal (Grossmann et al., 2016). Overall, the responses of each group to environmental factors differed due to their specific adaptation strategies.

## 5. Conclusion

The interspecies interactions and biogeography of MFW have been focused on a long-standing debate. This research tested three hypotheses of MFW in the oligotrophic tropical western Pacific Ocean. First, we found predominant linkages of MFW were different with depths. Second, we revealed biogeographic patterns of microeukaryotic communities largely matched with bacterial communities. Third, we suggested local environmental factors were more important than spatial factors in shaping the biodiversity and biogeographic of MFW, while NEC had a weak effect. Such knowledge is critical fundamental data for forecasting MFW changes in the face of future global change and future planning of marine protected areas.

## Data availability statement

The datasets presented in this study can be found in online repositories. The names of the repository/repositories and accession number(s) can be found at: https://www.ncbi.nlm.nih.gov/, PRJNA791001 https://www.ncbi.nlm.nih.gov/, PRJNA879911.

## Author contributions

QS and DS conceptualized the study and designed the experiment. QS performed the statistical analysis and wrote the manuscript with contributions from DS and CW. DS, CF, and YF participated in the research cruise and collected data. DS and CW approved the final version of the manuscript. All authors contributed to the article and approved the submitted version.

## Funding

This work was supported by the National Natural Science Foundation of China (42076122) and the China Ocean Mineral Resources Research and Development Association Program (DY-XZ-02; DY135-E2-3-04).

## Conflict of interest

The authors declare that the research was conducted in the absence of any commercial or financial relationships that could be construed as a potential conflict of interest.

## Publisher’s note

All claims expressed in this article are solely those of the authors and do not necessarily represent those of their affiliated organizations, or those of the publisher, the editors and the reviewers. Any product that may be evaluated in this article, or claim that may be made by its manufacturer, is not guaranteed or endorsed by the publisher.

## Footnotes

^1^ http://www.r-project.org
