# Supplementary material for: Microbial food webs share similar biogeographic patterns and driving mechanisms with depths in oligotrophic tropical western Pacific Ocean
Source: Front Microbiol. 2023 Jan 26;14:1098264. doi: 10.3389/fmicb.2023.1098264 (PMC9909095; doi:10.3389/fmicb.2023.1098264)
Supplement: Supplementary file 1 [file Data_Sheet_1.PDF]

Supplementary Material

Table S1. Summary statistics of sequence counts for each microeukaryotic sample before and after quality filtering.

Samples	Raw reads	High-quality reads	ASV numbers	Good's coverage (%)
S1-5	110348	88316	660	99.33
S2-5	97746	70524	846	99.07
S3-5	98920	68702	1318	97.51
S4-5	96299	64799	509	99.48
S5-5	109861	80068	888	98.67
S8-5	103056	80880	486	99.4
S1-75	109078	75944	526	99.31
S2-75	103514	72340	715	99.07
S4-75	98216	77976	605	99.54
S5-75	104007	70776	620	99.4
S6-75	105176	72998	579	99.01
S7-75	88519	68350	606	99.27
S8-75	96841	70865	628	99.16

S1-DCM	102735	52604	474	99.42
S2-DCM	105438	69475	628	99.24
S3-DCM	88817	28731	566	99.47
S4-DCM	95813	62204	380	99.46
S6-DCM	98686	54819	528	99.35
S7-DCM	94350	57183	420	99.52
S8-DCM	95105	28612	520	99.5
S1-200	108023	64306	415	99.53
S2-200	115360	21172	1010	99.08
S3-200	113828	21947	762	99.21
S4-200	96190	28702	789	99.18
S5–200	98944	25354	720	99.39
S6-200	109490	28473	596	99.36
S7-200	96135	40793	656	99.24
S8-200	106361	26371	844	99.25
S1-500	114165	67579	1288	97.36
S2-500	108634	70910	1065	97.89

S3-500	110093	65602	1166	97.81
S4-500	101445	59492	1156	97.75
S5-500	110740	67412	1226	97.34
S6-500	115544	73892	1091	97.96
S7-500	104699	85462	573	98.73
S8-500	100381	57484	1396	97.17
S2-3000	103313	66040	310	99.57
S3-3000	102244	57999	1312	97.29
S4-3000	110794	89415	176	99.76
S5-3000	108535	77009	230	99.72
S6-3000	107990	75003	199	99.72
S7-3000	112058	89829	277	99.77
S8-3000	109671	30960	292	99.77

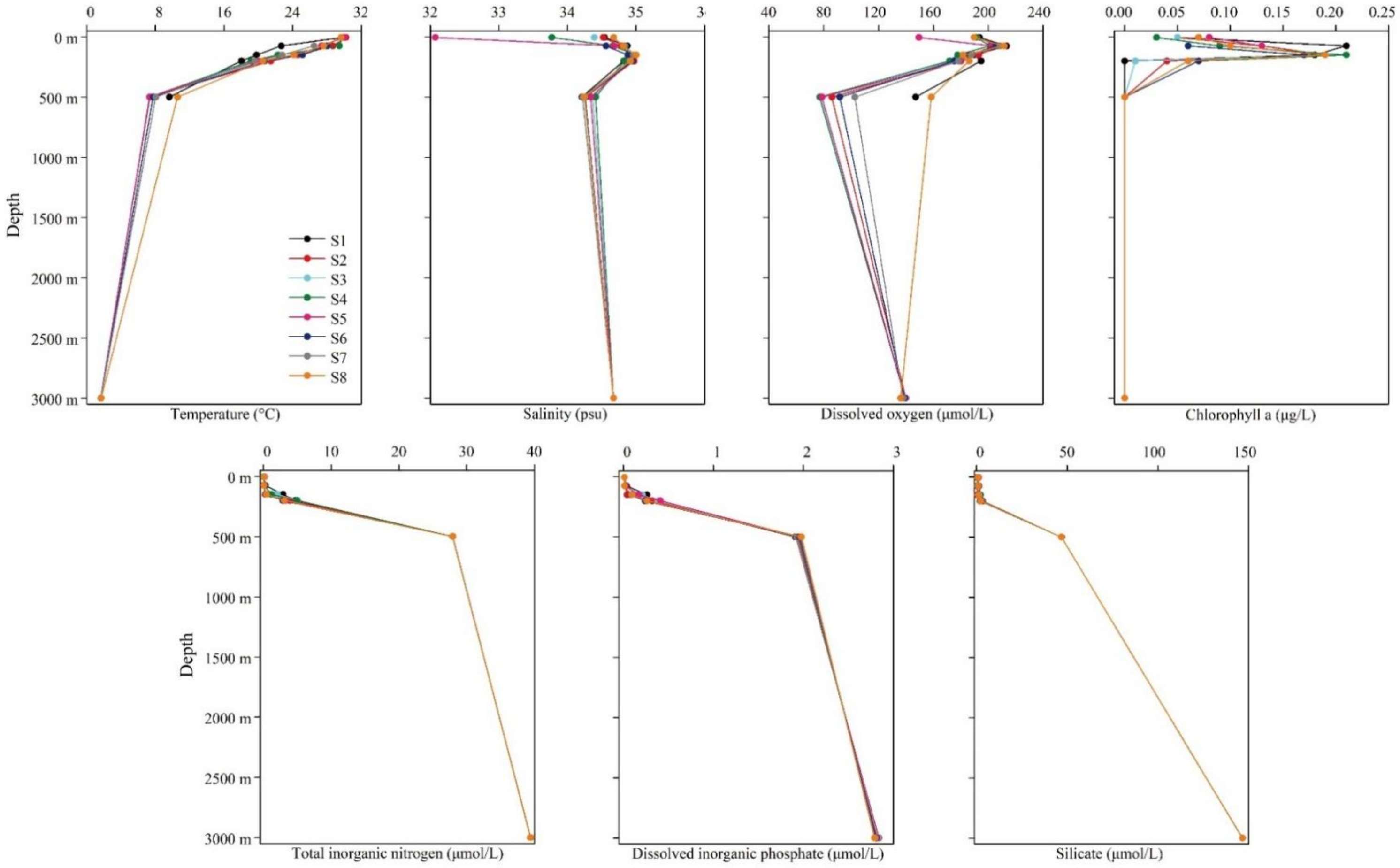

Fig. S1. Environmental factors of the water columns at different depths of eight sampling stations in the western subtropical Pacific Ocean.

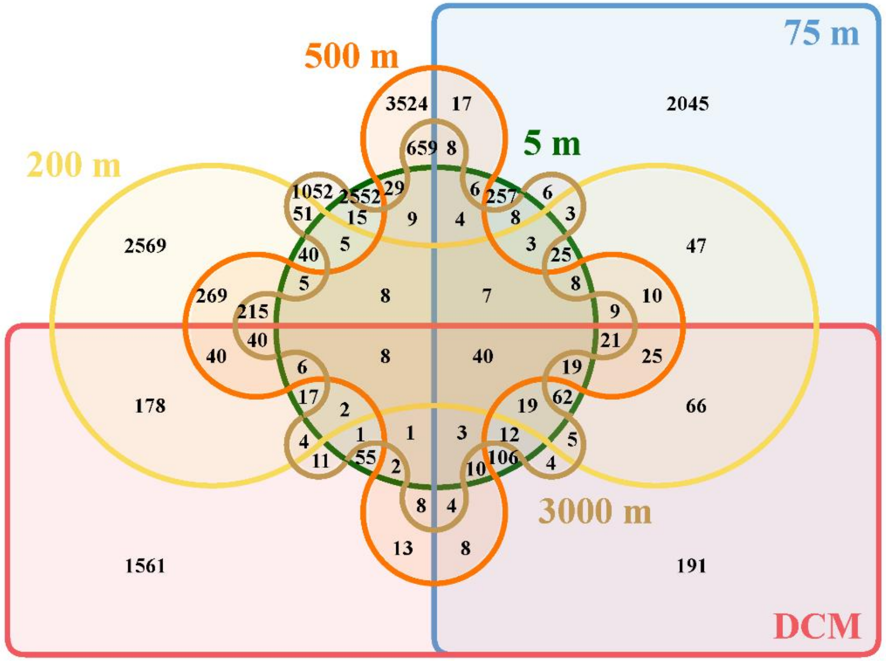

Fig. S2. Venn diagram showing the number of microeukaryotic ASVs that are unique and shared among six depth groups (5 m, 75m, DCM, 200 m, 500 m and 3000 m). DCM, denoted as deep chlorophyll a maximum layer.

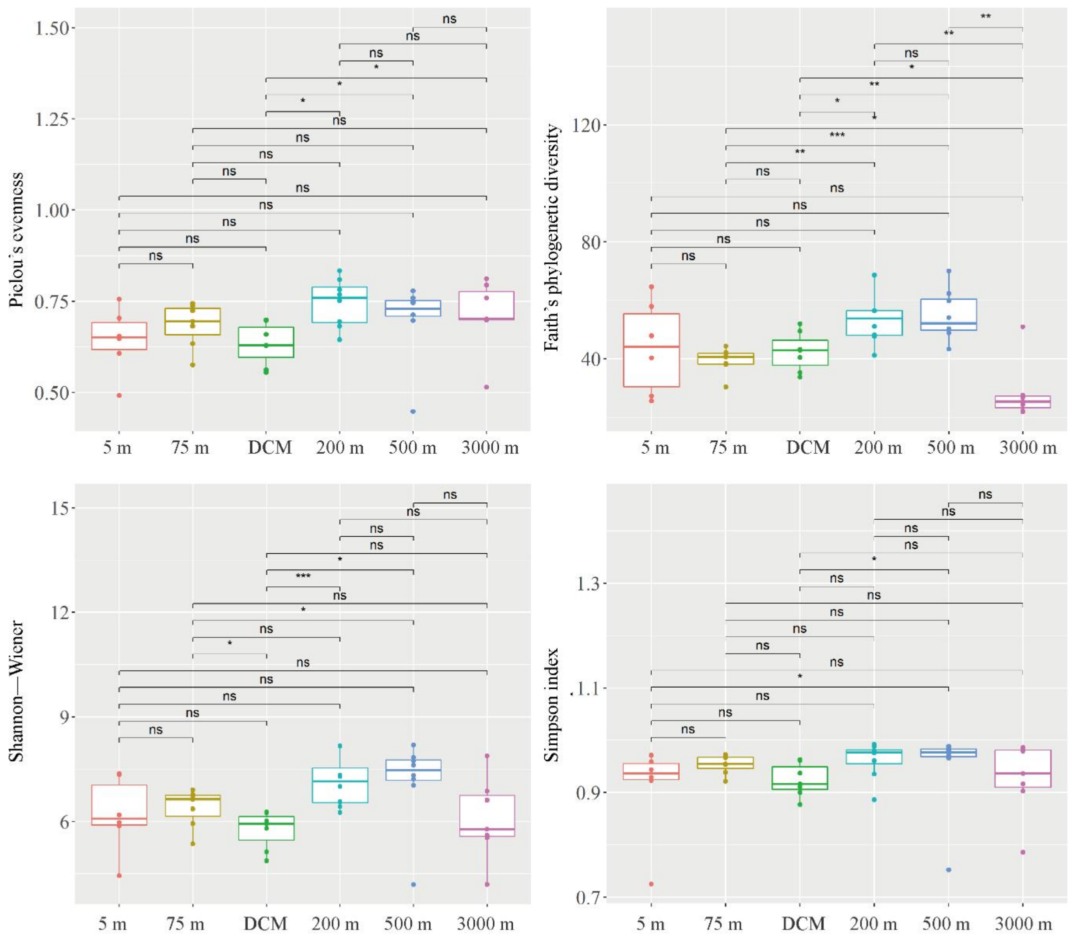

Fig. S3. Boxplots of microeukaryotic α-diversity indices in six depth groups (5 m, 75m, DCM, 200 m, 500 m and 3000 m), including Pielou's evenness, Faith's phylogenetic diversity, Shannon–Wiener and Simpson index. DCM, denoted as deep chlorophyll a maximum layer. Wilcox

tests were calculated on the α-diversity indices between depths. * represents a statistically significant difference of $P < 0.05$; ** represents a statistically significant difference of $P < 0.01$, *** represents a statistically significant difference of $P < 0.001$, ns represents no statistically significant difference.

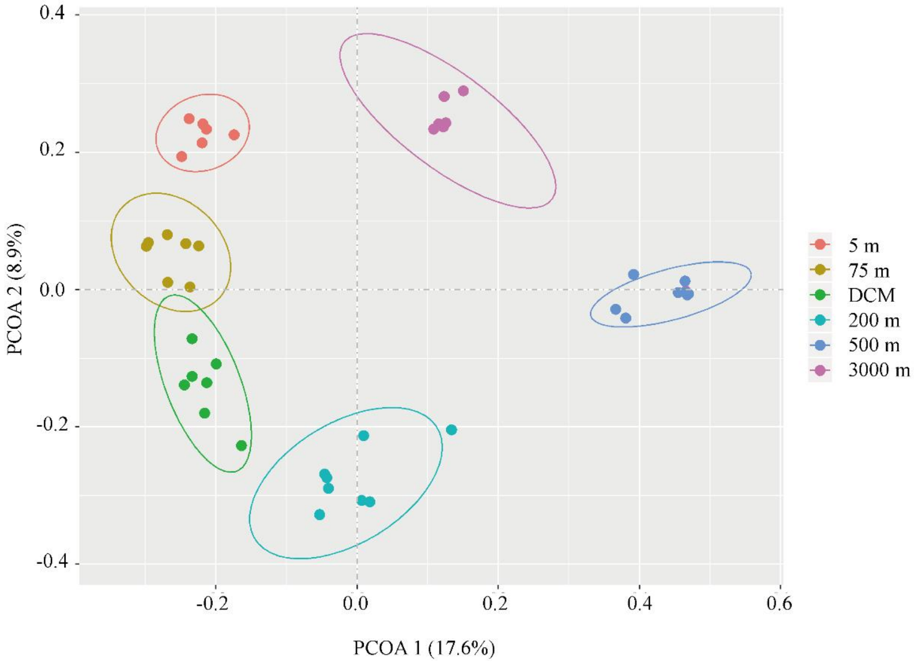

Fig. S4. Principal coordinates analysis (PCoA) plot based on the Bray-Curtis distance for microeukaryotic community in six depth groups.

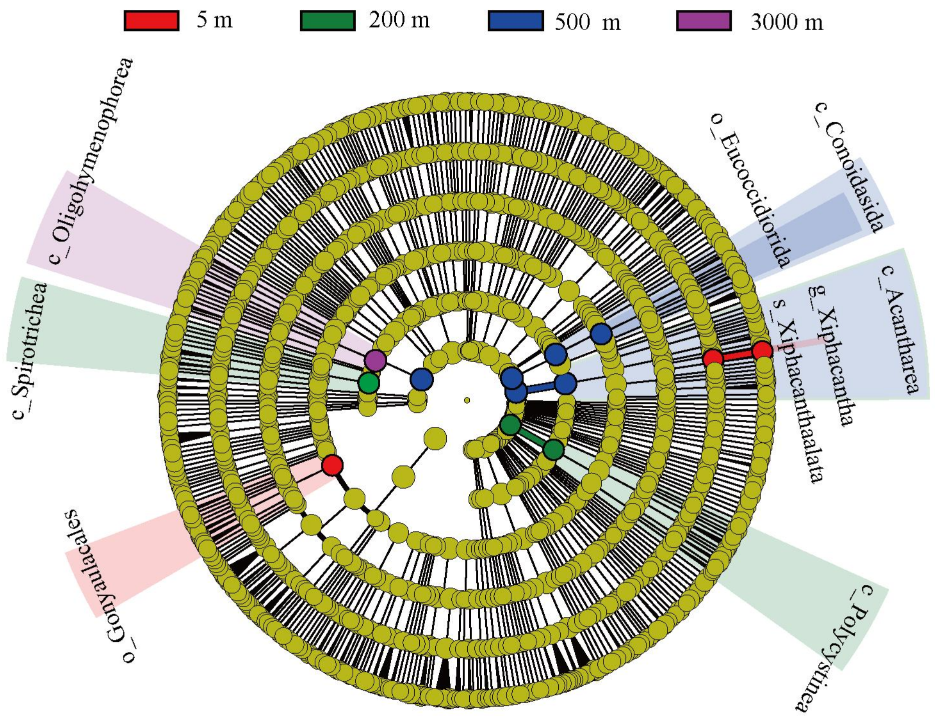

Fig. S5. Cladogram indicates the phylogenetic distribution of microeukaryotic lineages in six depth groups. The biomarkers for six depth groups are represented in the color (red indicate 5 m, green indicate 200 m, blue indicate 500 m and purple indicate 3000 m). Circles represent phylogenetic levels from phylum to genus inside out. c_: class; o_: order; g_: genus; s_: species.

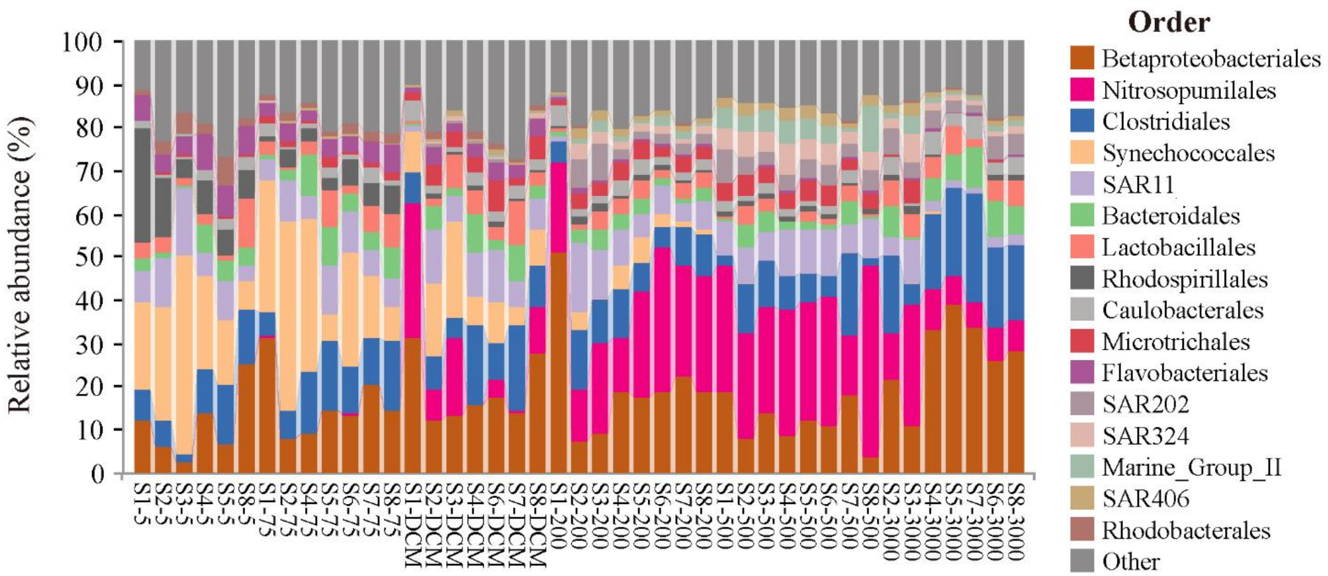

Fig. S6. Relative abundance of bacterial taxonomic groups at the order (relative abundance of > 1%) level across 43 samples.

Table S2. Quantitative effect of different stations and depths on variations in all, 5–200 m, and > 500 m bacteria and microeukaryotes based on permutational multivariate analysis of variance (PERMANOVA).

Factors	Depths		Stations		Sections	
	R^2	*P*	R^2	*P*	R^2	*P*

All bacteria	0.554	0.001	0.119	0.990	0.035	0.203
5–200m bacteria	0.406	0.001	0.222	0.891	0.066	0.121
> 500 m bacteria	0295	0.001	0.419	0.885	0.058	0.387
All microeukaryotes	0.397	0.001	0.144	0.958	0.031	0.262
5–200m microeukaryotes	0.302	0.001	0.251	0.639	0.056	0.160
> 500 m microeukaryotes	0.264	0.002	0.437	0.878	0.094	0.213

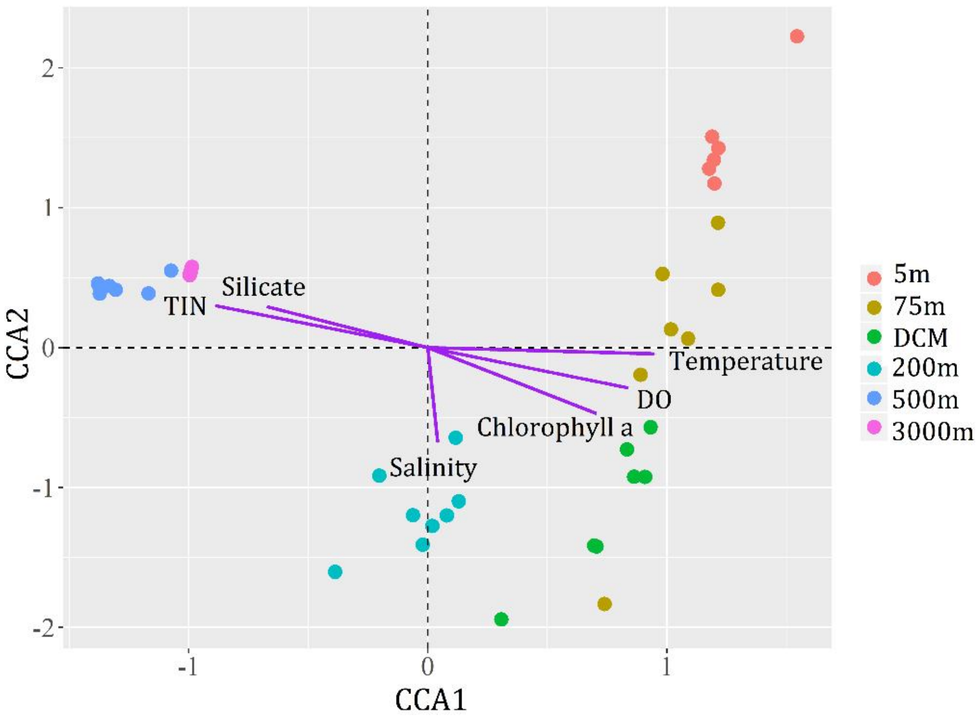

Fig. S7. CCA ordinations showing the microeukaryotic community composition in relation to environmental variable.

Table S3. Mantel tests for the correlations between spatial / environmental factors (Euclidean distance) and β-diversity of bacterial / microeukaryotic communities (Bray-Curtis dissimilarity) with 999 permutations. All, denoted as all microeukaryotes; 5–200 m, denoted as 5–200 m microeukaryotes; > 500 m, denoted as > 500 m microeukaryotes.

Factor	All bacteria		5–200 m bacteria		> 500 m bacteria		All microeukaryote		5–200 m microeukaryote		> 500 m microeukaryotes	
	ρ	*P*	ρ	*P*	ρ	*P*	ρ	*P*	ρ	*P*	ρ	*P*
All spatial factors	-0.084	0.999	-0.033	0.693	-0.061	0.693	-0.031	0.800	-0.003	0.476	-0.054	0.678
PCNM no.1	-0.032	0.865	-0.026	0.654	-0.014	0.403	0.004	0.360	0.021	0.265	0.017	0.321
PCNM no.2	0.122	0.060	0.112	0.049	-0.027	0.620	0.007	0.393	0.108	0.070	-0.069	0.621
PCNM no.3	-0.126	0.998	-0.121	0.998	0.034	0.342	-0.013	0.558	-0.079	0.845	0.026	0.362
PCNM no.4	-0.141	0.999	-0.094	0.972	0.103	0.201	-0.030	0.760	-0.139	0.986	0.130	0.192
PCNM no.5	0.003	0.450	0.078	0.106	-0.185	0.967	0.009	0.371	0.155	0.023	-0.148	0.856
All environmental factors	0.529	0.001	0.711	0.001	0.570	0.001	0.670	0.001	0.699	0.001	0.427	0.003
Temperature	0.592	0.001	0.780	0.001	0.628	0.001	0.711	0.001	0.734	0.001	0.480	0.004
Salinity	0.122	0.008	0.101	0.087	0.564	0.001	0.179	0.001	0.210	0.009	0.511	0.002
Dissolved oxygen	0.429	0.001	0.201	0.004	0.155	0.085	0.463	0.001	0.207	0.007	0.156	0.074
Chlorophyll a	0.333	0.001	0.305	0.001	/	/	0.414	0.001	0.281	0.002	/	/
Dissolved inorganic	0.517	0.001	0.746	0.001	0.618	0.001	0.635	0.001	0.656	0.001	0.553	0.002
Total inorganic nitrogen	0.518	0.001	0.702	0.001	0.618	0.002	0.634	0.001	0.613	0.001	0.553	0.001

Silicate	0.325	0.001	0.671	0.001	0.618	0.001	0.464	0.001	0.603	0.001	0.553	0.001
